# Deformation, microstructure and stiffness of the rat brain tissue during the development of experimental obstructive hydrocephalus

**DOI:** 10.1186/2045-8118-12-S1-O6

**Published:** 2015-09-18

**Authors:** Lauriane Jugé, Alice Pong, Andre Bongers, Ralph Sinkus, Lynne E Bilston, Shaokoon Cheng

**Affiliations:** 1 Neuroscience Research Australia, University of New South Wales, Australia; 2 Neuroscience Research Australia, Australia; 3 University of New South Wales, Australia; 4 King's College London, UK; 5 Neuroscience Research Australia, Macquarie University, Australia

## Introduction

Understanding neural injury in hydrocephalus and how key physiological factors changes during the course of the disease in-vivo remain unclear. This study aims to describe brain deformation, microstructural and mechanical properties changes during obstructive hydrocephalus (HCP) development in a rat model using multimodal magnetic resonance (MR) imaging.

## Methods

Hydrocephalus was induced in 8 Sprague-Dawley rats by injecting a kaolin suspension into the cisterna magna. 6 sham-injected rats were used as controls. MR imaging (9.4T, Bruker) was performed 1 day before, then at 3, 7 and 16 days post injection. T2-weighted MR images were collected to quantify ventricular enlargement and brain deformation. MR elastography was used to measure the brain stiffness, and diffusion tensor imaging (DTI) was conducted to observe brain tissue microstructure. Histology examination was performed immediately after the last MR scan. Generalized estimating equations (GEE) were used to assess linear relationships between variables while accounting for repeated measure design.

## Results

The enlargement of the ventricular system was associated with a decrease in the cortical gray matter thickness and basal ganglia cross-sectional area from day 3 (P<.001, for both), an alteration of the corpus callosum microstructure and rearrangement for the cortical gray matter microstructure (P<.001, for both), while a compression without gross microstructural alteration was evident for the basal ganglia and internal capsule (P<.001, for both), not seen in controls. Specifically, during hydrocephalus development, increase in space between the white matter tracts was observed in the corpus callosum (P<.001), while a decrease of space was observed for the internal (P<.001). For the cortical gray matter, an increase in extracellular tissue water originating from the ventricles via the discontinuous ependyma was observed and significantly associated with a decrease in stiffness (P=.001). Finally, for the basal ganglia, results suggested a progressive compression of the tissue, beginning with the periventricular region, then extending to the entire structure. The basal ganglia did not appear to be edematous, and the tissue compression was associated with an increase in stiffness (P=.001).

## Conclusions

This study outlines the temporal changes in tissue microstructure, water content and stiffness in different brain regions using DTI and MR elastography and how these changes are associated with ventricular enlargement. It shows that the effect of ventricular enlargement is not limited to periventricular white matter, severity of microstructural changes vary with brain region and there is regional and temporal variation in brain tissue stiffness during hydrocephalus development.

